# Identification of *E1-E4* allele combinations and ecological adaptability of soybean varieties from different geographical origins in China

**DOI:** 10.3389/fpls.2023.1222755

**Published:** 2023-09-04

**Authors:** Naiwen Zheng, Yukai Guo, Siyu Wang, Han Zhang, Li Wang, Yang Gao, Mei Xu, Wenyan Wang, Weiguo Liu, Wenyu Yang

**Affiliations:** ^1^ College of Agronomy, Sichuan Agricultural University, Chengdu, China; ^2^ Sichuan Engineering Research Center for Crop Strip Intercropping System, Sichuan Agricultural University, Chengdu, China; ^3^ Key Laboratory of Crop Ecophysiology and Farming System in Southwest China, Ministry of Agriculture, Chengdu, China

**Keywords:** soybean, photoperiod genotype, *E* gene, agronomic traits, KASP genotyping

## Abstract

The duration of soybean growth and development is regulated by *E1-E4* allele genes, which form the basis for ecological adaptation related to biomass accumulation, flowering and pod formation, maturation, and yield. To elucidate the effects of different combinations of *E1-E4* allele genes on soybean ecological adaptation, this study conducted competitive allele-specific PCR (KASP) analysis and photoperiod gene typing on 101 main soybean cultivars from different latitudes in China. The ecological adaptation of these cultivars in Sichuan was also investigated. The results showed that within a certain range (60-95 days), soybean varieties with a genotype combination of *E1/e2-ns/E3/E4* exhibited a longer growth period and demonstrated higher biomass and yield, displaying overall better performance. These varieties showed strong ecological adaptation in the Chengdu Plain region and are suitable for introduction in similar low to mid-latitude areas like the Chengdu Plain (30°N~32°N). Conversely, soybean varieties carrying a higher number of recessive alleles of *E1-E4* are not suitable for introduction in this region.

## 1 Introduction

Soybean (*Glycine max* (Linn.) Merr), originally from China, is currently cultivated worldwide and is an important grain and oil crop. In 2022, China’s soybean production reached 20.285 million tons, showing a 23.7% increase compared to the previous year. The Food and Agriculture Organization of the United Nations (FAO) predicts that the global population will reach 10 billion by 2050, necessitating a substantial increase in grain and oil production to meet future food demands. Soybean is a short-day plant that exhibits high sensitivity to photoperiod (Hadley et al., 1984). Photoperiodic response is one of the important factors influencing soybean’s regional adaptation and yield. Flowering marks the transition of soybean from the vegetative growth phase to the reproductive growth phase. The duration of growth and development is a crucial ecological trait for crops (Wang, 1981). Soybean varieties originating from different latitudes and environmental conditions exhibit varying photoperiod sensitivities. With the advancement of molecular biology techniques, molecular markers have become important tools for selecting target traits in crop varieties. Competitive allele-specific PCR (KASP) genotyping technology enables high-throughput screening of large populations and plays a significant role in gene mapping and germplasm diversity analysis (Grogan et al., 2016; Wu et al., 2018). Fang et al. applied the KASP genotyping technology to soybean photoperiod genes and used it to predict the adaptation regions of high-yielding soybean lines (Fang et al., 2021).

The growth period of soybean is mainly determined by factors that control its flowering and maturity (Bernard, 1971). The length of the growth period is the most fundamental characteristic for the formation of regional and seasonal types of soybean varieties (Hao et al., 1992). Studies by Han Tianfu et al. have shown that different soybean varieties exhibit significant differences in their growth period structure, but the proportion of flowering-podding stages (R1-R5) and filling stage (R6) in soybean varieties with the same growth period and growth period structure ratio also varies (Han et al., 1998; Wang et al., 2015). The entire growth period, vegetative growth period (V), reproductive growth period (R), and growth period structure (R/V) constitute the growth period traits of soybean, and selective breeding of growth period structure can improve the selection efficiency for soybean yield (Fehr, 1977; Sun et al., 1990). However, genetic variation in the main effect genes and quantitative trait loci (QTLs) that control delayed flowering and maturity enables soybean to have broad ecological adaptability (Garner and Allard, 1920). Based on relevant studies, it is known that the various allelic genes of *E1-E4* are the foundation for soybean flowering, maturity, and ecological adaptability. The soybean growth period gene *E1* is the first classic soybean maturity locus discovered, with the greatest impact on flowering and maturity periods, and all low-latitude varieties have *E1*. The *E2* gene has a small response to photoperiod, but a large contribution to growth period and stronger geographical adaptability, while *E3* and *E4* have significant correlations with photoperiod sensitivity. Under long day conditions, *E3* and *E4* have different sensitivities to R:FR, inducing the expression of *E1* and *E1L* (Watanabe et al., 2009; Jiang et al., 2014; Zhai et al., 2014; Kurasch et al., 2017). In natural environments, *E3* has a significant impact on flowering over a wide range of latitudes, while the impact of *E4* is limited to high latitude areas (Lu et al., 2015).

The flowering time determines the adaptability of soybean to different ecological regions. As a short-day photoperiod-sensitive crop, soybean is highly sensitive to the duration of sunlight, and the critical photoperiod of varieties decreases from high latitudes to low latitudes, resulting in a narrow ecological adaptability of each soybean variety (Fehr, 1987; Câmara et al., 1997; Agarwal et al., 2013). Under short-day conditions, photoperiod-insensitive varieties have early flowering and maturation, resulting in reduced yield (Spehar, 1995), while being adapted to high-latitude regions (Tsubokura et al., 2013). For example, soybean in high-latitude regions must flower under long daylight conditions in early summer and mature before autumn frost. However, in high-latitude regions, soybean flowering and maturation are often delayed. The growth period of soybean can be shortened by reducing sensitivity to long daylight. In low-latitude tropical and subtropical regions, high temperatures and short daylight promote rapid flowering and early maturity in photoperiod-insensitive soybean varieties, resulting in a short vegetative growth period and low yield. Therefore, delaying flowering is necessary in low-latitude regions to maintain a longer vegetative growth period and achieve high yield (Liu et al., 2008). Photoperiod genes play a crucial role in expanding the adaptability of soybean varieties to different latitude regions.

## 2 Materials and methods

### 2.1 Test materials

In this study, a total of 101 soybean (*Glycine max* (Linn.) Merr.) varieties from 21 provinces in 7 regions of China were collected for genotyping (**Table S1**).

### 2.2 Planting method

The 101 soybean varieties collected from 21 provinces in 7 regions of China were field-tested in 2021-2022 at Chongzhou (Sichuan Province, China) (30°33′N, 103°39′E), for phenotypic identification. The daily average sunlight duration during the growth period of the test site was about 13.76 h. These varieties were planted according to a randomized complete block design, with a row spacing of 0.5 m, a plant spacing of 0.1 m, and a distance of 0.5 m between plots with three replicates. The average temperature, sunlight duration, and precipitation in Chongzhou varied as shown in **Table 1**. From April to August, the average temperature during the entire soybean growth period was 1.3°C higher in 2022 than in 2021, especially in the later growth period of soybean from early July to late August, where the temperature was 2.3°C higher in 2022 than in 2021. The longest sunlight duration in mid-late June was 14.12 h, and decreased to 12.97 h in late August (**Table 1**).

**Table 1:** Changes in mean temperature, day and length precipitation in Chongzhou (2021-2022).

		Day Length (h)	Temperature (°C)		Precipitation (mm)	
			2021	2022	2021	2022
Apr.	Early	12.63	14.4	15.6	82.6	132.2
	Middle	12.95	15.9	15.1		
	Late	13.21	19.4	19.9		
May	Early	13.48	21.0	20.1	147.8	216.4
	Middle	13.71	20.8	18.7		
	Late	13.91	21.1	21.5		
June	Early	14.05	21.8	24.1	64.2	103
	Middle	14.12	23.1	24.8		
	Late	14.12	25.1	27.8		
July	Early	14.06	25.4	27.5	331.9	13.8
	Middle	13.94	25.7	26.8		
	Late	13.75	26.8	26.7		
Aug.	Early	13.52	27.4	28.2	45.3	126.6
	Middle	13.26	24.8	30.6		
	Late	12.97	22.6	26.7		

### 2.3 Agronomic traits determination

The whole growth period of soybean was recorded based on the identification method proposed by Fehr and Carviness (Fehr, 1977). The flowering period is from emergence (VE) to beginning of flowering (R1), and the maturation period is from emergence (VE) to initial maturity (R7). Three plants were selected for individual plant determination in each plot, and dynamic changes in plant height were measured weekly. For the full pod stage (R6), five plants were selected in each plot for measurement of plant height and aboveground biomass. At full maturity (R8), five plants were selected in each plot for measurement of yield traits such as single plant grain weight and effective pod number.

2.4 DNA extraction

Soybean DNA was extracted using a plant genomic DNA extraction kit (Solarbio, Beijing, https://www.solarbio.com). The OD_260/280_ and DNA concentration were measured using a micro-volume spectrophotometer (GE GeneQuant 100, General Electric Company, Boston, https://www.ge.com). DNA samples with OD_260/280_ ratios of 1.7-2.0 were selected and diluted to a concentration of 50-100 ng/μL with ddH_2_O.

### 2.4 *E1-E4* genotyping and identification

The photoperiodic SNP markers and KASP genotyping primers (**Table S2**) were designed based on the study by Liu et al (Liu et al., 2020). The designed primers need to add a different linker sequence (FAM: 5’ GAAGGTGACCAGTTCATGCATGCT 3’ and HEX: 5’ GAAGGTCGGAGTCAACGGATT 3’) to enable identification by fluorescent probes labeled with FAM and HEX, respectively, and to produce fluorescent signals during PCR amplification. We performed using the Zhonghuang 13 (*E1/e2- ns/E3/E4*) and Zhonghuang 30 (*e1-as/E2/E3/E4*) (Liu et al., 2020; Lu et al., 2020) were used as positive controls during the KASP amplification process.

KASP amplification was performed using a real-time PCR instrument QuantStudio 6 Flex (Thermo Fisher Scientific, Massachusetts, https://corporate.thermofisher.com). For the KASP analysis, the mixed primers included 12 μL (100 μM) of each allele-specific forward primer, 30 μL (100 μM) of a common reverse primer, and 46 μL of ddH_2_O. Each 10 μL KASP reaction contained 1 μL of genomic DNA (50-100 ng/μL), 0.14 μL of the mixed primers, 5 μL of HiGeno 2x Probe Mix (Jasongen Biotech, Beijing, https://www.jasongen.com), and 4 μL of ddH_2_O. The amplification was carried out using the AQP™ genotyping system in a PCR cycling program: pre-denaturation at 95°C for 10 min, followed by 10 cycles of denaturation/annealing (95°C for 20 s, annealing temperature decreased from 65°C to 55°C by 0.6°C per cycle for 40 s), and then 34 additional cycles (95°C for 20 s, 55°C for 40 s). The fluorescent endpoint reading was performed at 35°C for 30 s.

### 2.5 Statistical analysis

OriginPro 2022b (https://www.originlab.com) and IBM SPSS Statistics 26.0 (https://ibm.com/products/spss-statistics) were used to perform analysis of variance (ANOVA) on different allele combinations of *E1-E4* and agronomic traits. Based on the genotype results, conduct a comprehensive evaluation of different photoperiod gene types, soybean plant type traits, yield, and reproductive period structure using principal component analysis, cluster analysis, and other methods.

#### 2.5.1 Comprehensive evaluation value


(1) $$y_{1}=\sum_{i=1}^{n}\left(z_{i}\times \frac{x_{1i}}{\sqrt{a_{1}}}\right)\;\;i=1,2,\cdots ,n\;$$

(2) $$y_{2}=\sum_{i=1}^{n}\left(z_{i}\times \frac{x_{2i}}{\sqrt{a_{2}}}\right)\;\;i=1,2,\cdots ,n\;$$

(3) $$y=b_{1}\times y_{1}+b_{2}\times y_{2}$$

In the formula, α_1_ and α_2_ are the initial eigenvalues of the first and second components in principal component analysis, x_1i_ and x_2i_ represent the component matrix values of each trait indicator for components 1 and 2, z_i_ is the standardized value of each sample trait indicator, b_1_ and b_2_ are the variances (%) of the initial eigenvalues of components 1 and 2, and y_1_ and y_2_ are the scores of each sample for components 1 and 2, respectively. y represents the comprehensive score of each sample’s principal components.

## 3 Results and analysis

### 3.1 Genotyping of soybean photoperiodic *E1-E4* genes using KASP

**Figure 1:**
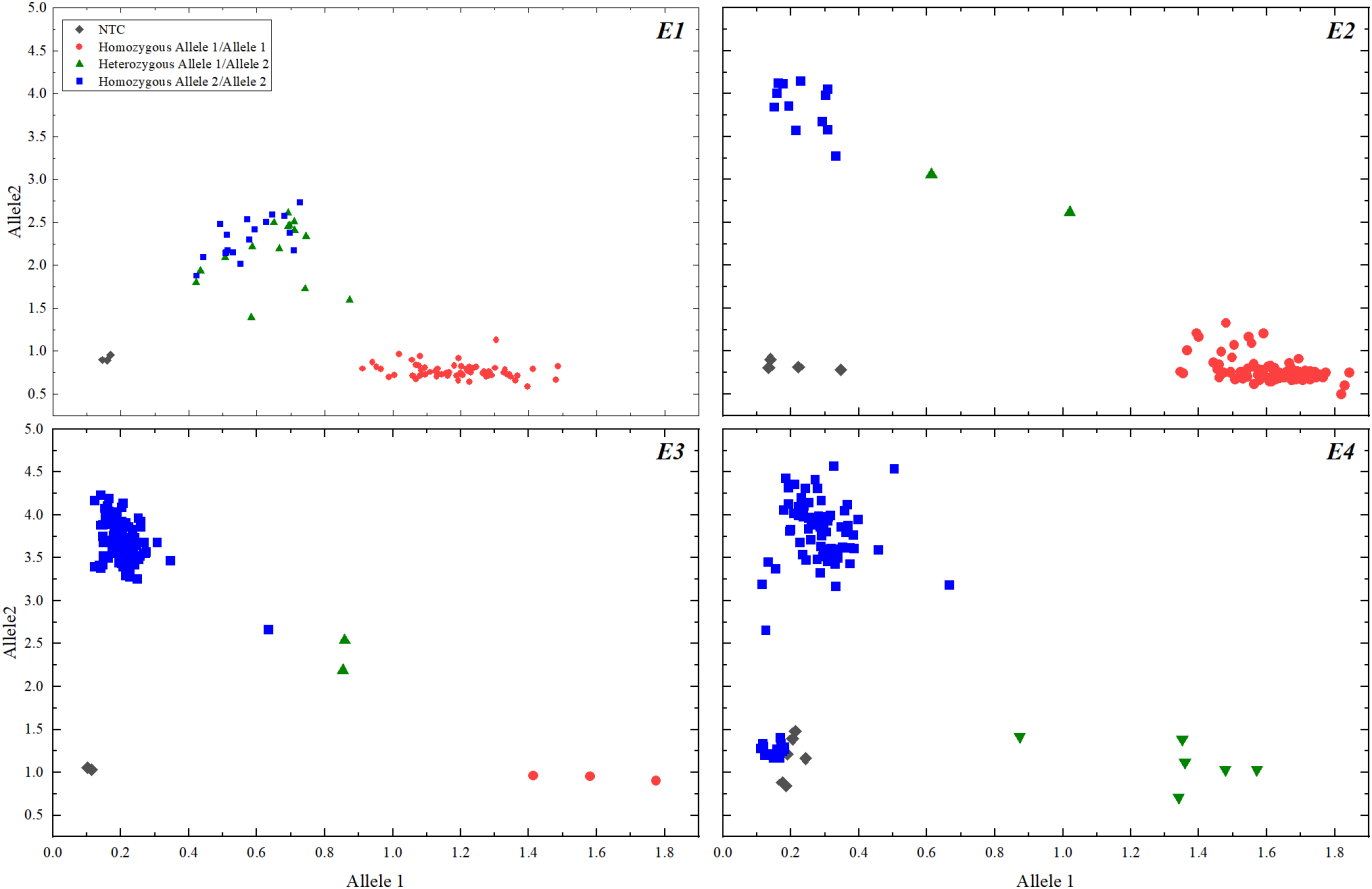
Allelic Discrimination.

All 101 soybean varieties were genotyped for *E1-E4* using KASP, and the genotyping results are shown in **Figure 1**. The photoperiodic genotypes of soybean varieties Zhonghuang 13 and Zhonghuang 30 used as positive controls were previously identified as *E1/e2-ns/E3/E4* and *e1-as/E2/E3/E4*, respectively.

After KASP genotyping, the photoperiodic genotyping results of 101 soybean varieties from different regions of China were obtained (**Table S1**). According to **Table S1**, 74 soybean varieties had a photoperiodic genotype of *E1/e2-ns/E3/E4*, 7 varieties had a genotype of *e1-as/E2/E3/E4*, 11 varieties had a genotype of *e1-as/e2-ns/E3/E4*, 1 variety had a genotype of *e1-as/e2-ns/e3-tr/E4*, 2 varieties had a genotype of *E1/e2-ns/e3-fs/E4*, and 6 varieties had a genotype of *E1/E2/E3/E4*.

### 3.2 Comparison of soybean germplasm resources’ reproductive period in different regions

**Figure 2:**
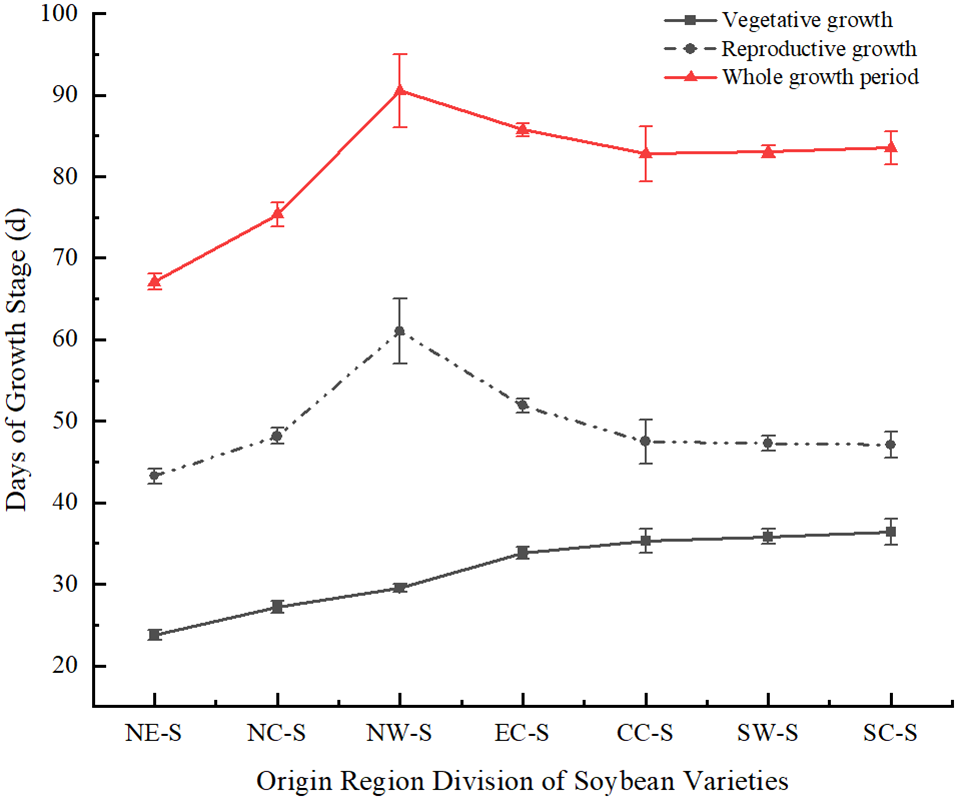
Comparison of soybean growth period in different regions under monocropping. NE-S, Northeast Soybean; NC-S, North China Soybean; NW-S, Northwest Soybean; EC-S, East China Soybean; CC-S, Central China Soybean; SW-S, Southwest soybean; SC-S, South China soybean.

Soybean varieties from seven different latitude regions, including Northeast, North China, East China, Central China, Northwest, Southwest, and South China, were introduced to Sichuan for spring planting. The average latitude of the seven regions was ranked as follows: Northeast > North China > Northwest > East China > Central China > Southwest > South China. The reproductive growth period of these soybean varieties was longer than their vegetative growth period. Among them, soybean varieties from Northeast region, which had the highest latitude when introduced to Sichuan, had both shorter vegetative and reproductive growth periods compared to varieties from other regions. Southern soybean and southwestern soybean have longer vegetative growth periods, while northwestern soybean has a shorter vegetative growth period but a longer reproductive growth process. The overall growth period of northwestern soybean (from emergence to physiological maturity) is the longest (**Figure 2**). During the vegetative growth phase, the duration increases as the latitude of the soybean origin decreases. In the overall growth process of soybean, most of the durations decrease as the latitude of the soybean origin decreases, showing a consistent trend with the reproductive growth process.

### 3.3 Flowering maturity and geographic distribution of *E1-E4* allele combinations in different soybean varieties

**Figure 3:**
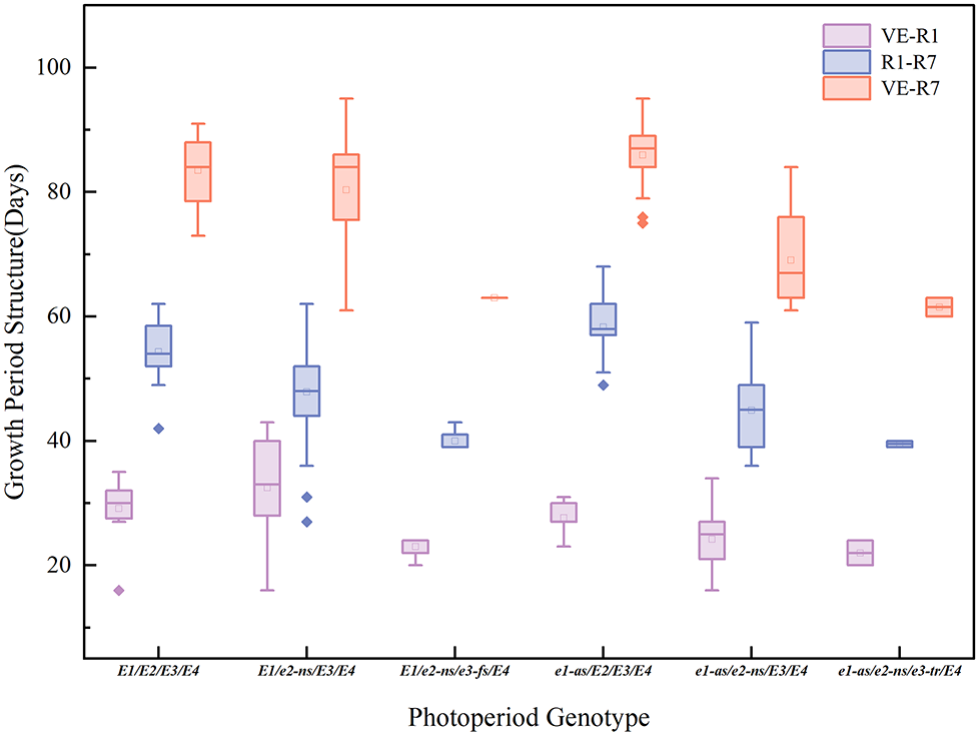
Growth period structure (vegetative growth period: days from VE to R1; reproductive growth period: days from R1 to R7) and maturity time (days from VE to R7) of different photoperiod genotypes.

The *E* gene is the main genetic factor that determines the growth period of soybeans (Li et al., 2017). We assessed the phenotypic and growth period characteristics of 101 cultivated soybean varieties in Chongzhou based on their combinations of *E1-E4* alleles (**Figure 3**; **Table 2**). During the years 2021-2022, varieties carrying the *e1-as/e2-ns/e3-tr/E4* and *e1-as/e2-ns/E3/E4* allele combinations exhibited the shortest flowering and maturation times, while those carrying the *E1/e2-ns/E3/E4* and *E1/E2/E3/E4* allele combination had the latest flowering time. Varieties with the *e1-as/E2/E3/E4* allele combination had the longest podding and maturation times. The *E1/e2-ns/E3/E4* allele combination had the smallest growth period structure ratio, while the *e1-as/E2/E3/E4* and *e1-as/e2-ns/E3/E4* allele combination had the largest growth period structure ratio.

**Table 2:** Flowering and maturity time of soybean genotypes with different photoperiods.

Photoperiod Genotype	Vegetative Growth Period (d)	Reproductive Growth Period (d)	Maturity time (d)	Growth period structure (R/V)
*E1/E2/E3/E4*	29.17 ± 4.84	54.33 ± 5.52	83.5 ± 5.95	1.95 ± 0.62
*E1/e2-ns/E3/E4*	32.50 ± 6.67	47.84 ± 6.56	80.34 ± 9.67	1.54 ± 0.40
*E1/e2-ns/e3-fs/E4*	25.00 ± 1.81	43.33 ± 7.84	68.33 ± 8.63	1.74 ± 0.30
*e1-as/E2/E3/E4*	27.64 ± 2.65	58.29 ± 5.17	85.93 ± 6.04	2.12 ± 0.26
*e1-as/e2-ns/E3/E4*	22.64 ± 6.12	45.27 ± 3.38	67.91 ± 5.75	2.14 ± 0.57
*e1-as/e2-ns/e3-tr/E4*	22.00 ± 2.83	39.50 ± 0.71	61.50 ± 2.12	1.81 ± 0.27

Based on the combined results of the two years of field experiments, varieties carrying a higher number of recessive *E1-E4* allele combinations exhibited shorter flowering and maturation times. However, no consistent pattern was observed between the growth period structure ratio and the photoperiod genotype.

Furthermore, we conducted analysis of variance to investigate the effects of *E1-E4* genes on flowering and maturation times in soybean. Due to the presence of only dominant alleles for *E4* in all cultivated varieties, variance analysis was not performed for the *E4* gene. Based on the combined field data from two years, we observed that both the *E1* and *E2* genes collectively influenced the soybean growth period. Specifically, *E1* and *E3* significantly influenced soybean flowering time, with *E1* exerting a greater effect than *E3*. Moreover, both *E1* and *E3* had highly significant impacts on soybean maturation time. However, the effect of *E2* on soybean maturation time was greater than that of *E1* (**Table 3**).

**Table 3:** Analysis of ANOVA of E for flowering and maturity time (days).

Flowering				Maturity			
Factor	DF	Mean Square	F Value	Factor	DF	Mean Square	F Value
*E1*	1	1377.609	34.742***	*E1*	1	850.864	8.516**
*E2*	1	174.930	3.831	*E2*	1	952.352	9.581**
*E3*	2	203.555	4.551*	*E3*	2	859.493	8.948***
*E1:E2*	1	1247.317	30.948***	*E1:E2*	1	3118.293	35.206***
*E1:E3*	1	155.269	3.393	*E1:E3*	1	629.876	6.235*
*E2:E3*	1	405.777	9.116**	*E2:E3*	1	1715.987	17.953***

*P ≤ 0.05; **P ≤ 0.01; ***P ≤ 0.001.

**Figure 4:**
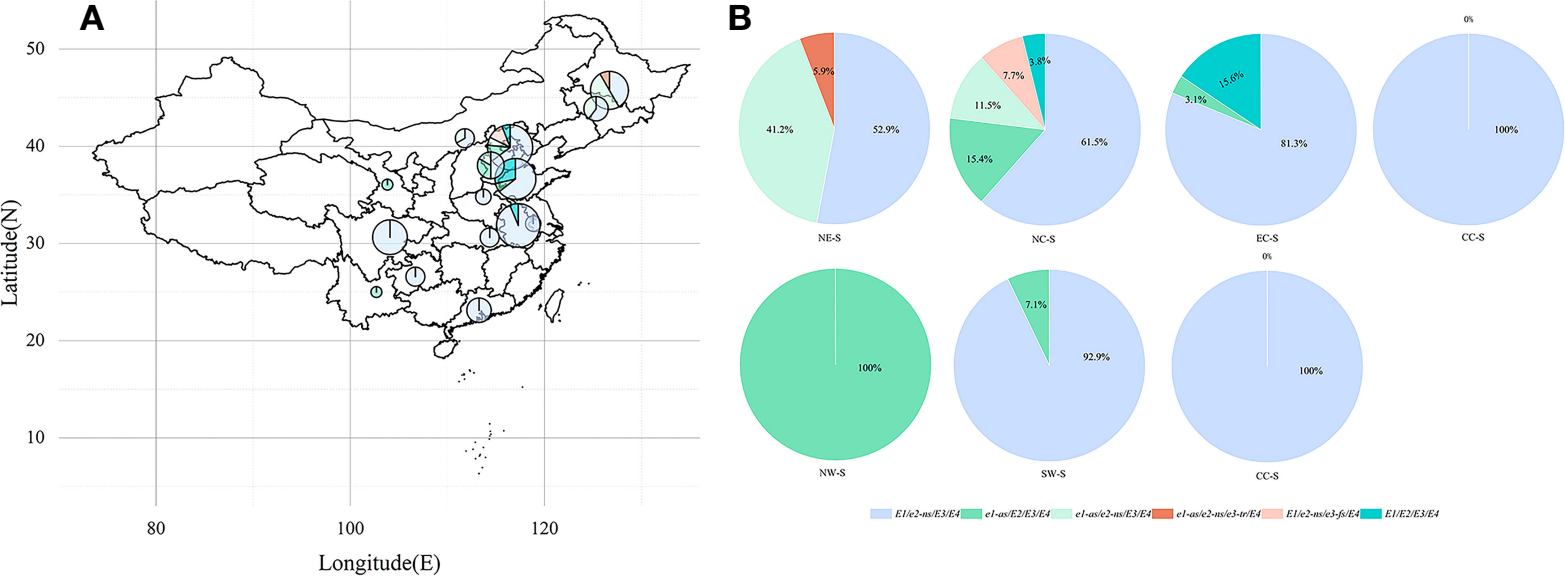
Geographical distribution of *E1-E4* allele combinations in China. **(A)** Each pie chart shows the proportional distribution of the *E1-E4* allele combination in its province (autonomous region). The size of the pie chart represents the number of varieties originating in each province (autonomous region). **(B)** Each pie chart shows the proportional distribution of the region of origin where the *E1-E4* alleles are located.

We analyzed the geographic distribution of cultivated soybean varieties from 15 provinces (autonomous regions) in China (**Figure 4**). The vast majority of soybean varieties in most provinces (autonomous regions) carry the *E1/e2-ns/E3/E4* allele combination (**Figure 4A**). In the Northeast region, there are *E1/e2-ns/E3/E4*, *e1-as/e2-ns/E3/E4*, and e*1-as/e2-ns/e3-tr/E4*, while *e1-as/e2-ns/E3/E4* and *e1-as/e2-ns/e3-tr/E4* are mainly distributed in this region (**Figure 4B**), with Heilongjiang province having the largest proportion (**Figure 4A**). In the North China region, the *E1-E4* allele combinations are the most diverse, with Beijing having the widest distribution. In the East China region, *E1/e2-ns/E3/E4*, *e1-as/E2/E3/E4*, and *E1/E2/E3/E4* are distributed in this area, with the vast majority of *E1/E2/E3/E4* distributed in this region (**Figure 4B**). In the Central and Southern China regions, the main allele combination is *E1/e2-ns/E3/E4*, while in the Northwest region, it is mainly *e1-as/E2/E3/E4*, and in the Southwest region, it is mainly *E1/e2-ns/E3/E4* and *e1-as/E2/E3/E4*.

### 3.4 Agronomic traits of *E1-E4* allele combinations of different soybean varieties

**Figure 5:**
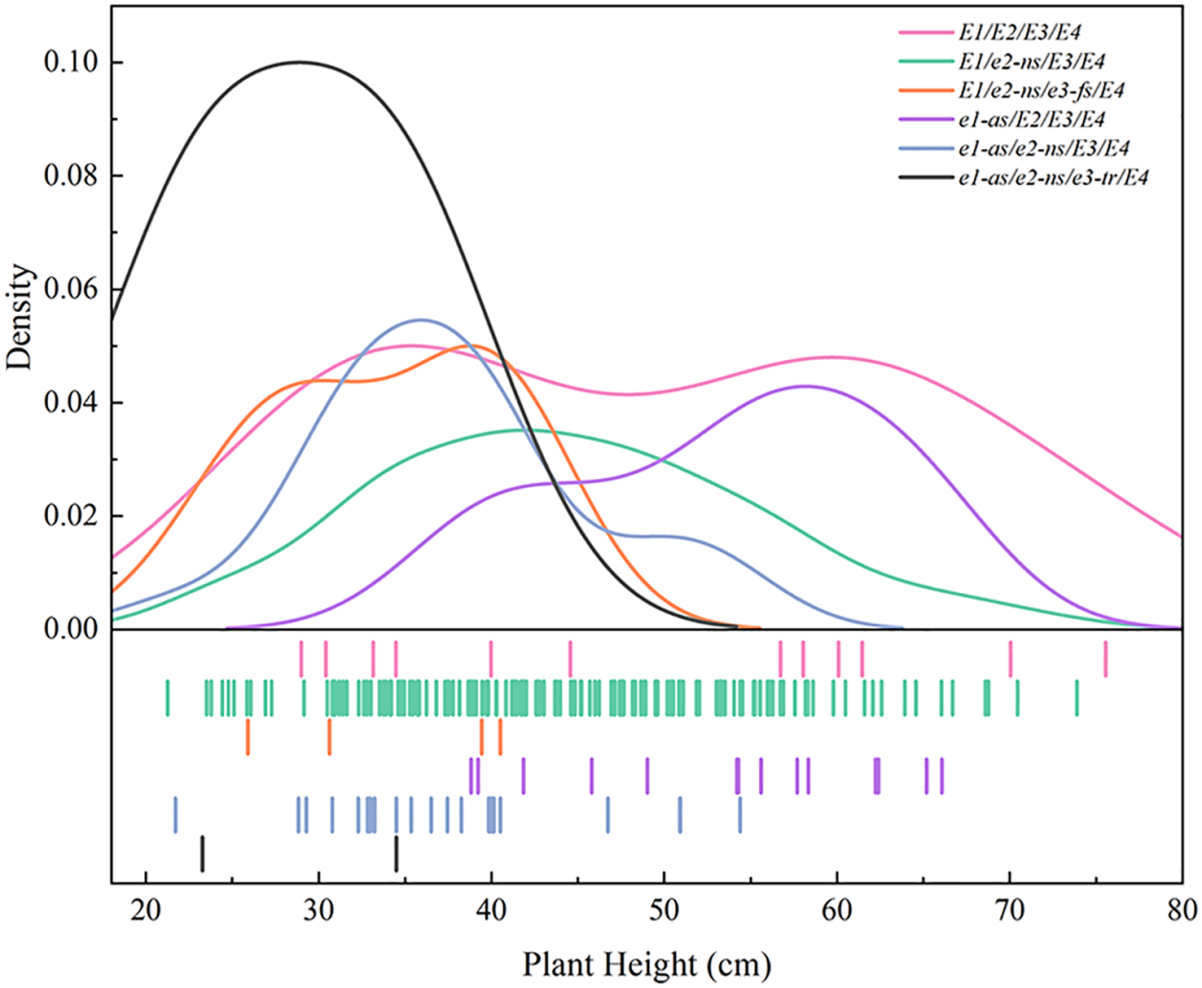
Plant height distribution of *E1-E4* allele combinations of different soybean varieties.

Based on the comprehensive analysis of two years of field experiments, we observed that the height distribution of the 101 cultivated soybean varieties fell within the range of 21 to 80 cm (**Figure 5**), respectively. Among the investigated soybean varieties, those with the genotype *E1/e2-ns/E3/E4* exhibited the widest height distribution, with the highest density of plants observed in the 30-60 cm range (**Figure 5**). In contrast, plants with the genotype *E1/E2/E3/E4* showed a more dispersed height distribution, ranging from 28 cm to 76 cm. Plants with the genotype *E1/e2-ns/e3-fs/E4* had a height of approximately 30-40 cm, while those with the genotype e*1-as/E2/E3/E4* exhibited a height distribution ranging from 38 cm to 67 cm. The *e1-as/e2-ns/E3/E4* genotype displayed a height distribution between 21 to 55 cm, with a primary concentration between 32 to 41 cm. Finally, plants with the genotype *e1-as/e2-ns/e3-tr/E4* exhibited heights of 34.5 cm and 23.3 cm in the two respective years, respectively. Overall, soybean varieties carrying more recessive alleles of *E1-E4* exhibited lower plant heights, while those carrying more dominant alleles of *E1-E4* exhibited higher plant heights.

**Figure 6:**
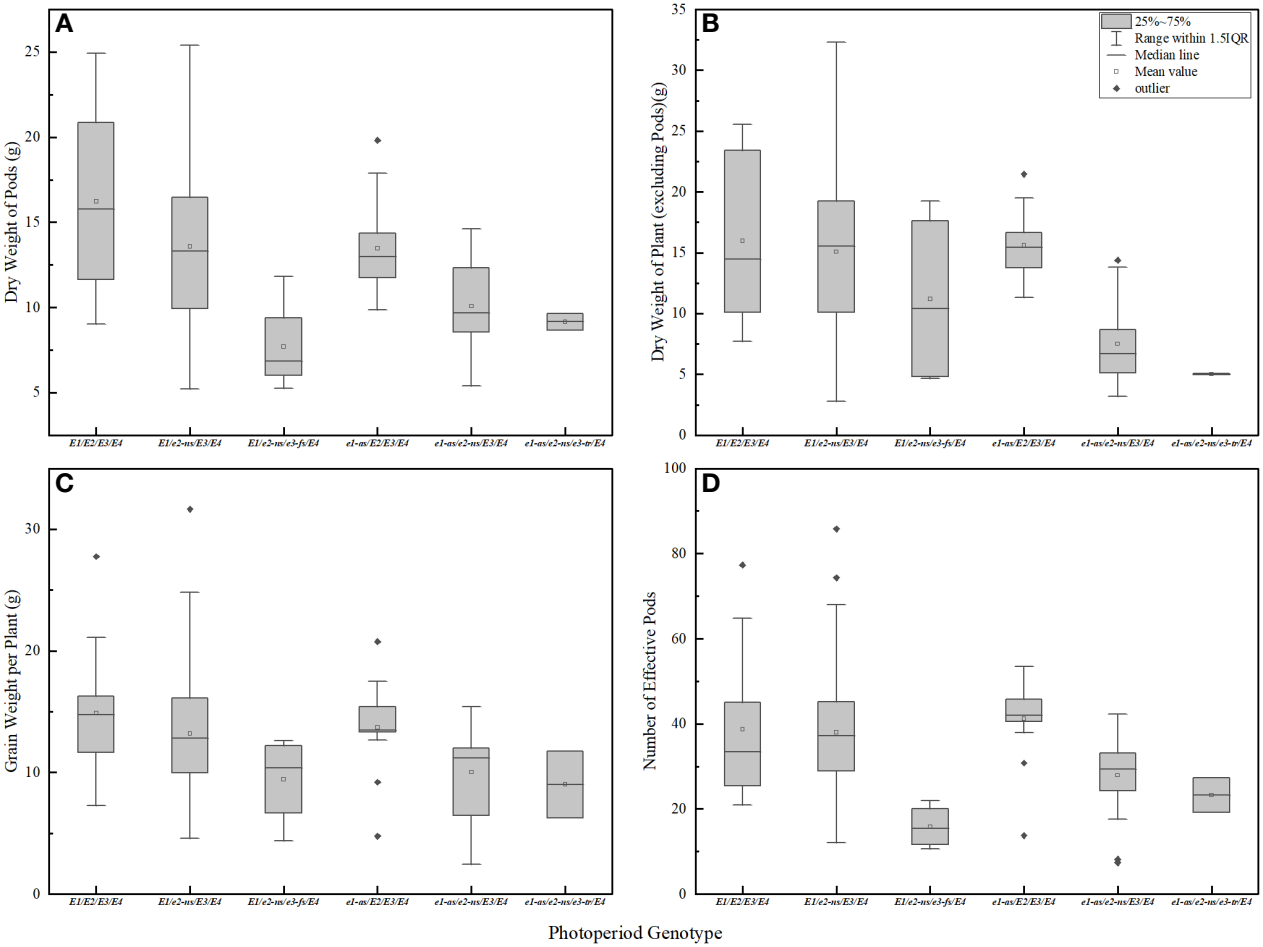
Biomass and yield of soybean with different photoperiod genotypes. **(A)** pod dry weight in R6 period; **(B)** plant dry weight (except pod) in R6 period; **(C)** effective pod number per plant in R8 period; **(D)** grain weight per plant in R8 period.

The genotype of photoperiodic genes is closely related to soybean pod biomass and yield per plant. Soybean varieties with a higher frequency of dominant alleles *E1-E4* have more effective pods and higher yield per plant. Soybean varieties with mutated alleles of *E1-E4* have fewer pods, lower pod biomass, lower final yield per plant, and lower total dry matter (**Figure 6**). Among the genotypes, *E1/E2/E3/E4* exhibited the highest pod biomass (**Figure 6A**), while *E1/e2-ns/E3/E4* and *e1-as/E2/E3/E4* showed higher stem, leaf, and branch biomass (**Figure 6B**). In contrast, *E1/e2-ns/e3-fs/E4* had the lowest pod biomass, and *e1-as/e2-ns/e3-tr/E4* displayed the lowest stem, leaf, and branch biomass. Regarding yield components, *E1/E2/E3/E4* displayed the highest individual seed weight, whereas *e1-as/e2-ns/e3-tr/E4* had the lowest individual seed weight (**Figure 6C**). Furthermore, *e1-as/E2/E3/E4* exhibited the highest number of effective pods per plant, while *E1/e2-ns/e3-fs/E4* displayed the lowest (**Figure 6D**). Consequently, soybean varieties carrying a higher number of recessive *E1-E4* gene combinations exhibited lower yield and biomass.

In addition, we also conducted a variance analysis of the effects of *E1-E4* on soybean agronomic traits (**Table 4**). The results show that plant height of various cultivated soybean varieties is influenced by the *E2* and *E3* gene, with *E2* having a greater effect on plant height than *E3*; The accumulation of biomass in soybean was jointly affected by *E1* and *E3*, where stem, leaf, and branch biomass, as well as pod biomass, were primarily influenced by the *E1* gene. Notably, the influence of *E1* on pod biomass and total biomass accumulation was greater than that of *E3*. Additionally, *E1-E4* genes significantly influenced soybean yield components. Specifically, the *E3* gene significantly affected the formation of effective pods in soybean, while the *E1* gene had a significant impact on grain weight per plant.

**Table 4:** Analysis of ANOVA of *E* for agronomic traits.

Factor	DF	Plant Height	Dry weight of plant (excluding pods)	Dry weight of pods	Total biomass	Number of effective pods	Grain weight per plant
*E1*	1	0.420	16.670***	8.882**	16.605***	4.598*	5.298*
*E2*	1	13.696***	1.775	3.694	3.058	2.189	2.596
*E3*	2	3.461*	2.403	4.102*	3.378*	6.797***	1.853
*E1:E2*	1	11.413***	34.104***	14.848***	32.050***	14.256***	11.453***
*E1:E3*	1	3.602	3.923*	1.661	3.596	2.169	1.384
*E2:E3*	1	6.664*	3.621	8.090**	6.471*	13.162***	3.714

*P ≤ 0.05; **P ≤ 0.01; ***P ≤ 0.001. The values in the table are F-Values of one-way ANOVA.

### 3.5 Comprehensive evaluation of different soybean photoperiod genotypes and growth period structure, plant type and yield

The data on the developmental structure (VE-R1, VE-R7), plant morphology (plant height, stem diameter), and yield traits (single seed weight, number of effective pods, pod dry weight, stem and leaf dry weight) of 101 soybean varieties were standardized using the Z-score method. The suitability of factor analysis was tested using the KOM and Bartlett’s sphericity tests (**Table 5**). The KOM value was 0.778, and the Bartlett’s sphericity test result was 400.718 with a Sig value of 0.000. Both tests indicated that there was a certain correlation between the various indicators of the developmental structure, plant morphology, and yield traits, and therefore, all indicators were suitable for factor analysis.

**Table 5:** Principal Component Analysis Results.

Total Variance Explained						
Component	Initial Eigenvalues			Extraction Sums of Squared Loadings		
	Total	of Variance (%)	Cumulative (%)	Total	of Variance (%)	Cumulative (%)
1	4.012	50.153	50.153	4.012	50.153	50.153
2	1.359	16.984	67.136	1.359	16.984	67.136
3	0.821	10.260	77.396			
4	0.614	7.669	85.065			
5	0.422	5.275	90.340			
6	0.384	4.803	95.143			
7	0.244	3.044	98.186			
8	0.145	1.814	100.000			

**Table 5** showed that the first two principal components explained 67.136% of the total variance, indicating that the two principal components extracted could represent 67.136% of the original eight indicators, including developmental structure, plant morphology, and yield traits. According to the coefficients of the two principal components in **Table 6**, it was found that the coefficients of stem and leaf dry weight, maturity time (VE-R7), flowering time (VE-R1), plant height, pod dry weight, and number of effective pods in principal component Y1 were larger than those of other variables, indicating that principal component Y1 was a comprehensive reflection of these six trait indicators. In principal component Y2, the coefficients of stem diameter and single seed weight were larger than those of other variables, indicating that principal component Y2 was mainly reflected by these two trait indicators.

**Table 6:** Component Matrix.

Trait Indicator	Component	
	1	2
Dry weight of plant (excluding pods)	0.861	0.218
VE-R7	0.857	-0.059
VE-R1	0.828	-0.061
Plant height	0.750	-0.394
Dry weight of pods	0.711	-0.047
Number of effective pods	0.682	-0.193
Grain weight per plant	-0.024	0.790
Stem thick	0.562	0.697

Based on the principal component analysis, a comprehensive score was given to the eight trait indicators. Soybean varieties with higher scores were mainly from the eastern and northern regions of China, mainly in the Yellow-Huaihe region, with the *E1/e2-ns/E3/E4* gene combination. These varieties had higher biomass, yield, and longer developmental periods. The comprehensive scores of soybean varieties with more *E1* and *E2* recessive alleles were generally lower. Most soybean varieties from the northeastern region had lower comprehensive scores. This method can be used to screen for ideal soybean varieties suitable for different geographical cultivation patterns.

## 4 Discussion

### 4.1 *E1-E4* allele combinations of different soybean varieties regulate their growth period structure

Soybeans carrying different allele combinations of *E1-E4* genes exhibit variations in flowering and maturation time. Chongzhou (30°33′N, 103°39′E) is located in the southwestern region of China, and the majority of the cultivated soybean varieties used in this experiment originate from areas north of Chongzhou, where daylight duration exceeds that of the experimental site. Previous studies have demonstrated significant interactions between major genes controlling the growth period of soybeans, as well as interactions between genes and photoperiod, which directly influence soybean’s growth and development. Soybeans regulate their flowering in response to changes in day length by perceiving the duration of the photoperiod (Garner and Allard, 1920). *E1* expression is suppressed under short-day conditions, leading to prolonged flowering time under long-day induction. Similarly, *E2* affects soybean maturation time under short-day conditions (Wang et al., 2008; Thakare et al., 2010; Xia et al., 2012; Thakare et al., 2011). In this study, we confirmed that *E1* has a greater effect on the flowering and maturity of soybean, and its fertility period is generally shorter than that of the source, but *E3* also has a greater effect on the structure of the fertility period of soybean, and the effect is greater than that of *E2* (**Table 3**); and the previous study found that the *E1-E3* genes have a greater effect on the flowering time and maturity time of soybeans, but the effect of *E3* is smaller than that of *E1* and *E2* (Liu et al., 2020). *E1* has a highly significant effect on the length of flowering time of soybean, more than 80% of cultivated soybean varieties originating from latitudes higher than the Chengdu Plain, the length of sunshine is relatively shortened, so the flowering time of soybean is shortened under short sunshine conditions. *E2* had a large effect on the different stages of soybean growth and development (**Table 2**). Soybean varieties containing the *e2-ns* allele exhibit smaller R/V values (1.54-2.14), significantly lower than those containing the *E2* allele (R/V values of 1.95-2.12). Under spring planting conditions, recessive *E1* and *E3* genes promote soybean flowering, while recessive *E1-E3* genes shorten the reproductive growth period (R1-R7) and reduce the R/V value. Moreover, a higher number of recessive genes in the genetic combination leads to a certain degree of shortening in various growth stages. In the background of *e2-ns/e3-tr/E4* or *e2-ns/e3-fs/E4* gene combinations, varieties carrying recessive *E1* or *E1* genes exhibit early flowering and maturation. However, in the background of *e2-ns/E3/E4*, soybean varieties containing the *E1* gene exhibit delayed flowering and maturation compared to *e1-as*. This conclusion is consistent with previous studies (Liu et al., 2020). Under the *E2/E3/E4* background, soybean varieties have longer reproductive growth periods and maturation times. Compared to *e1-as*, soybean varieties containing the *E1* gene have shorter reproductive growth periods, but the difference in maturation time is not significant.

### 4.2 *E1-E4* allele combination affects the ecological adaptability of soybean

Relevant studies have shown that the *E3* and *E4* genotypes not only regulate the flowering period of soybeans but also determine plant architectural traits such as the number of main stems, pod development, and stem growth after flowering, all of which are closely associated with yield (Xu et al., 2013). Additionally, the progression of the soybean growth period directly impacts plant architectural traits. The diversity of *E1-E4* allele combinations leads to variations in plant architectural traits and agronomic characteristics, including yield-related factors. In this study, we found that *E1* had some effect on soybean yield composition both on soybean pod formation was significant, and the effect of *E2* and *E3* genes on plant height of introduced soybeans was significant, and the effect of *E2* was greater than that of *E3* (**Table 4**). Previous research has revealed that varieties carrying the *E2/E3* combination can produce a greater number of main stems and flowers while maintaining a consistent pod set ratio, thereby resulting in increased pod production (Kawasaki et al., 2018). We also found that *E1* and *E3* significantly affected the composition of soybean plant biomass, with *E1* being the genetic locus that mainly affected the biomass of each component of soybean. Varieties carrying a higher number of recessive *E1-E4* alleles exhibited lower plant height and biomass in the Chongzhou region, while those with a greater number of dominant *E1-E4* alleles displayed higher levels of these agronomic traits. This can be attributed to the effects of *E1-E3* on soybean flowering and maturation time, with *E1* specifically prolonging the flowering (vegetative growth) period. The extension of the vegetative growth phase in soybeans is advantageous for increased flower and leaf production, thereby promoting reproductive growth (Cober et al., 2005; Zhang et al., 2007).

Among the different genotypes, the agronomic traits (plant height, biomass, and yield) of the *e1-as/e2-ns/e3-tr/E4* combination exhibited the lowest levels. Therefore, based on a comprehensive evaluation of genotypes and agronomic traits, the soybean variety VE-R7, characterized by a longer growth period within the range of 60-95 days and the genomic combination of *E1/e2-ns/E3/E4*, displayed higher biomass and yield. It exhibited strong ecological adaptability in regions with moderate latitudes, making it suitable for cultivation in areas similar to the Chengdu Plain, characterized by relatively lower latitudes. Within the same growth period range of 60-95 days, soybean varieties carrying a higher number of recessive *E1-E4* alleles primarily originated from high latitude regions, and their agronomic traits exhibited the lowest levels, rendering them unsuitable for cultivation in regions with relatively lower latitudes.

## 5 Conclusion

The various alleles of *E1-E4* genes serve as the foundation for soybean flowering, maturation, and ecological adaptation, with mutations in the *E1-E4* loci leading to early flowering and maturation. The growth period, yield traits, stress resistance, and ecological adaptability are relevant factors to consider in germplasm introduction, and the degree of ecological differences between the introduction region and the source region is crucial for successful introduction. Within a certain range (60-95 days), soybean varieties with the genotype *E1/e2-ns/E3/E4* and longer growth periods, known as VE-R7, exhibit higher biomass and yield, demonstrating overall better performance. They also demonstrate strong ecological adaptability in the Chengdu Plain region, making them suitable for introduction in similar low- to mid-latitude areas (30°N~32°N), such as the Chengdu Plain. However, caution should be exercised when introducing soybean varieties carrying multiple recessive alleles of *E1-E4* in this region to avoid potential issues of early flowering and maturation, which could result in reduced yields.

## Data availability statement

The original contributions presented in the study are included in the article/**Supplementary Material**. Further inquiries can be directed to the corresponding author.

## Author contributions

Conceptualization; NZ. Methodology; NZ, MX and WW. Writing original draft preparation; NZ. Software; NZ and YKG. Data curation; NZ and YKGu. Investigation; NZ, YKG, SW and HZ. Experiment; NZ, YKG, LW and YG. Supervision; WL and WY. All authors have read and agreed to the published version of the manuscript.
